# Geographical mapping and seroprevalence of *Burkholderia pseudomallei* amongst livestock species in Lao People’s Democratic Republic

**DOI:** 10.1371/journal.pntd.0012711

**Published:** 2025-02-12

**Authors:** Suwei Zheng, James R. Young, Syseng Khounsy, Phouvong Phommachanh, Peter Christensen, Watthana Theppangna, Tom Hughes, Adisone Temmerath, Alex Inthavong, Phoummavanh Inthapanya, Sivone Panyasith, Koukeo Phommasone, Direk Limmathurotsakul, Elizabeth A. Ashley, Stuart D. Blacksell, Michael P. Ward

**Affiliations:** 1 Sydney School of Veterinary Science, The University of Sydney, Camden, New South Wales, Australia; 2 Mahidol-Oxford Tropical Medicine Research Unit (MORU), Faculty of Tropical Medicine, Mahidol University, Bangkok, Thailand; 3 National Animal Health Laboratory, Vientiane, Lao People’s Democratic Republic; 4 Lao-Oxford-Mahosot Hospital-Wellcome Trust Research Unit (LOMWRU), Mahosot Hospital, Vientiane, Lao People’s Democratic Republic; 5 Centre for Tropical Medicine and Global Health, Nuffield Department of Medicine, University of Oxford, Oxford, United Kingdom; Tufts Medical Center, UNITED STATES OF AMERICA

## Abstract

The Gram-negative bacterium *Burkholderia pseudomallei* causes a severe infectious disease known as melioidosis in humans and animals. It is considered endemic in tropical countries, including Thailand, Lao PDR (Laos), and Northern Australia. *B. pseudomallei* is a saprophyte found in contaminated soil and surface water. Humans and animals can become infected via direct exposure to contaminated water or soil and inhalation of dust or water droplets. Despite the high morbidity and mortality rates of melioidosis, there is a lack of knowledge of its geographical distribution and seroprevalence, even within endemic countries, raising a significant public health concern. For a better understanding of melioidosis in livestock in Laos, both as an animal health concern and as an indicator of human risk, we collected serum samples from an abattoir monitoring program for *B. pseudomallei* antibody testing using the Indirect Haemagglutination Assay (IHA). Out of the 917 sera collected, major findings included the identification of a significant cluster (p = 0.041) in the southwest border region adjoining northeastern Thailand, in the province of Savannakhet in Laos. Sera collected in January 2020 had the highest *B. pseudomallei* seroprevalence (17.0%), and cattle had the highest seroprevalence (22.8%), followed by buffalo (19.7%) and swine (4.0%). The *B. pseudomallei* seroprevalence results among the common livestock species and the maps generated can assist with future monitoring, prevention, and detection of melioidosis in Laos.

## Author summary

Melioidosis is a potentially fatal disease caused by the bacterium *Burkholderia pseudomallei*, which is commonly found in soil and water in tropical regions. Both animals and humans can become infected through direct contact with contaminated sources, and the disease is endemic in countries like Laos, Thailand, and northern Australia. However, there is limited knowledge about its spread among animals, especially in Laos. This study focused on mapping the geographical distribution and seroprevalence (the presence of antibodies) of *B. pseudomallei* in livestock in Laos. Serum samples from buffalo, cattle, and swine were collected from slaughterhouses and tested for antibodies against *B. pseudomallei*. Our findings revealed that *B. pseudomallei* exposure was widespread among livestock, with cattle showing the highest seroprevalence, followed by buffalo and swine. The highest rates of exposure were found in the southern provinces, especially Savannakhet, which shares a border with northeastern Thailand. These results suggest that livestock could serve as indicators of human risk for melioidosis and highlight the need for ongoing surveillance and monitoring of the disease in both animals and humans. The study provides valuable data to support future efforts in preventing, detecting, and managing melioidosis in Laos.

## 1. Introduction

Melioidosis is characterised by sepsis, pneumonia, and abscess formation in almost any organ caused by *Burkholderia pseudomallei*. This soil-dwelling Gram-negative bacterium exists as a saprophyte in soil and surface water in tropical and subtropical areas between 20°N and 20°S with endemic hotspots in Northern Australia and Southeast Asian countries [1–5]. Recently, more regions of endemicity have been uncovered in Africa, the Pacific, and the Americas [1].

Melioidosis affects both humans and animals [1]. It is associated with high morbidity of around 21% in Australia [6], with a case fatality rate of up to 50% in humans reported [7]. The study by Limmathurotsakul et al. [3] in Thailand between 2006–2010 estimated a morbidity rate of 1.63, 0.02, and 0.01 per 100,000 per year in goats, pigs and cattle, respectively. Furthermore, it is assumed that both humans and animals acquire the disease in similar ways [5] through direct exposure to contaminated water or soil, direct inoculation in skin abrasions and inhalation of dust or water droplets [1,3,8]. In addition, studies have suggested possible human-to-human, animal-to-animal and animal-to-human transmissions, although environmental transmission is the likely source of most infections [3,5]. The range of potential transmission pathways indicates the significance of melioidosis as an emerging disease of significant public health concern. It emphasises the need for better recognition and understanding of the geographical distribution of *B. pseudomallei* throughout the tropics to support disease prevention and control efforts.

There has been an increased recognition of melioidosis in animals [9,10]. Studies have reported infection in a wide variety of species with a wide range of clinical manifestations and susceptibilities; sheep and goats are particularly susceptible, with the highest prevalence rate in livestock species [3], followed by pigs which are mostly asymptomatic [11], and lastly, cattle and water buffalo that are relatively resistant to infection despite their constant exposure to mud [12]. Geographically, animal melioidosis is most notably recognised in Thailand, with high morbidity rates in the northeast [3].

In the Lao People’s Democratic Republic (Lao PDR or Laos), there is no surveillance system for animal melioidosis, and there has only been one reported case of animal melioidosis confirmed by the Lao-Oxford-Mahosot Hospital-Wellcome Trust Research Unit (LOMWRU) [13]. This highlights how little is known about the geographical distribution of animal melioidosis, where in most rural areas, there is limited access to microbiological facilities [7]. Human cases have mainly been reported from Vientiane and southern provinces; however, there is a lack of diagnostic microbiology in many parts of the country, so there is the potential for reporting bias [13].

There have been outbreaks of human melioidosis in non-endemic regions recently, demonstrating the capability of *B. pseudomallei* to occur in non-endemic areas with zoonotic infections, global trade, and animal migratory patterns [1]. Overall, melioidosis holds a significant public health concern due to its high morbidity and mortality rates in humans and livestock and its potential zoonotic and saprozoonotic nature. Significant gaps in knowledge of epidemiology remain even within endemic countries. Livestock could act as sentinels for human infection, so livestock surveys and monitoring can also benefit public health within a One Health framework. To better understand animal melioidosis, data were obtained from a survey conducted in Laos in which serum samples collected from an abattoir monitoring program were randomly selected to test for melioidosis using Indirect Haemagglutination Assay (IHA). IHA measures detectable antibodies against *B. pseudomallei* [14]. IHA is not recommended as a disease diagnostic test due to its moderate specificity (about 72%) and low sensitivity (about 55%) [15,16]; however, it can be used as a serological test evaluating exposure to *B. pseudomallei*, particularly in areas where endemicity of melioidosis is low or unknown [17].

This study and data analysis aimed to map the geographical distribution of *B. pseudomallei* seroprevalence and compare the seroprevalence in swine, buffalo, and cattle. Our goal is to assist with monitoring the distribution and occurrence of *B. pseudomallei* in Laos and surrounding countries, supporting early implementation, prevention, detection, and intervention of this under-recognised emerging disease.

## 2. Materials and methods

### Ethics statement

An animal ethics approval for this survey was obtained from the National Animal Health Laboratory Institutional Review Board, Ministry of Agriculture and Forestry, Department of Livestock and Fisheries, Lao PDR; approval number 0019/DLF.

### 2.1 Sampling frame and sample selection

Between 2019 and 2020, the US-DTRA-funded programme undertook a surveillance project that collected samples from buffalo, cattle, and swine at abattoirs within Lao districts. Samples were collected each month before slaughter between May 2019 and November 2020, and 976 buffalo, 2,053 cattle and 2,161 swine serum samples were obtained. Serum samples were stored at the National Animal Health Laboratory (NAHL) in Vientiane. The available list of samples was used as the study sampling frame. Simple randomisation and systematic selection was applied: every 10^th^ sample was selected for testing using the Indirect Haemagglutination Assay (IHA). Seroprevalence was defined by species: swine, buffalo, and cattle, with the target location set to be Lao PDR. Due to resource constraints and the number of available test kits, approximately 900 samples were targeted for testing.

### 2.2 Indirect haemagglutination assay

An Indirect Hemagglutination Assay (IHA) was used to detect antibodies against *B. pseudomallei* in serum samples (MEDKIT, Bangkok, Thailand. The test was performed according to the manufacturer’s instructions and is briefly outlined below. The serum was first inactivated by incubating for 30 minutes in a 56°C water bath. 25 µl of the inactivated serum was transferred to a microcentrifuge tube with 225 µl of 5% uncoated RBC added, then mixed thoroughly and incubated for 30 minutes at room temperature. The microcentrifuge tube was centrifuged for 10 minutes at 2,000 rpm. The serum was diluted 1:10 to use as absorbed serum for testing. Twenty-five microliters of diluent buffer was added to wells in columns 2 to 10 of a 96 U well microtitre plate, followed by 25 µl of absorbed serum added to wells in columns 1 and 2. A two-fold serial dilution was performed from columns 2 to 10, with 25 µl discarded from the final column, resulting in serum dilutions ranging from 1:10 to 1:5,120. Twenty-five microliters of melioidosis test cells were added to wells in columns 1 to 10, making the final serum dilutions range from 1:20 to 1:10,240. Serum control was established by adding 25 µl of absorbed serum and 25 µl of negative control cells in column 11, while cell control was made by adding 25 µl of diluent buffer and 25 µl of *B. pseudomallei* test cells in column 12. The plate was gently tapped to mix, incubated in a humid box for 2 hours at room temperature, and then read and recorded by technicians. A cut-off value of 1:320 was used for positive results. Each plate of 96 wells can run six samples plus a negative and positive control. Negative wells have no red cell agglutination with an intact button at the bottom of the well. Positive wells have red cell agglutination, with red cells settled as a fine carpet or appearing as a loose button with ragged or folded edges. The titre recorded is the first positive well. An adjacent tube was used if a sample had low volume or was of poor quality.

### 2.6 Mapping tool

**Fig 1:**
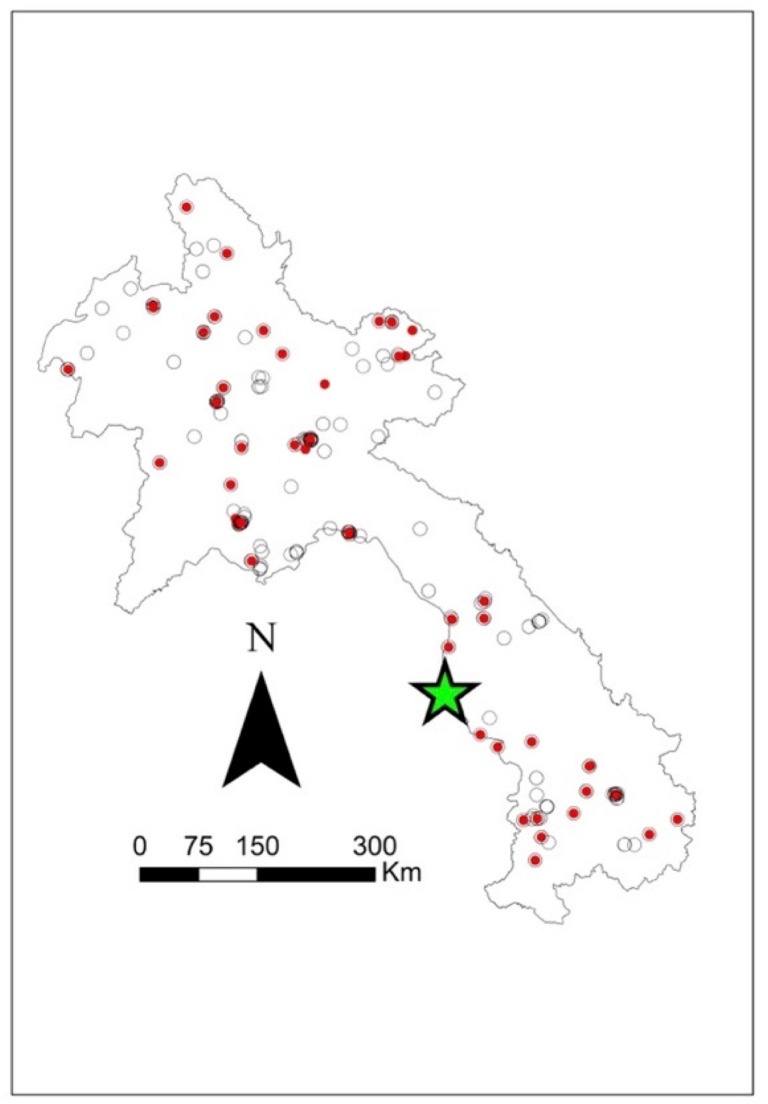
Geographical distribution of all *B. pseudomallei* seroprevalence amongst the livestock species (swine, buffalo, and cattle) tested using IHA in 2019–2020. Closed red circles represent seropositive. Open circles represent seronegative. The green star represents a significant cluster (p = 0.041) at 16.572049 N, 104.768658 E via SaTScan v 9.6. The scale bar and north arrow are included. Map shapefiles were sourced from https://diva-gis.org/data.html.

**Fig 2:**
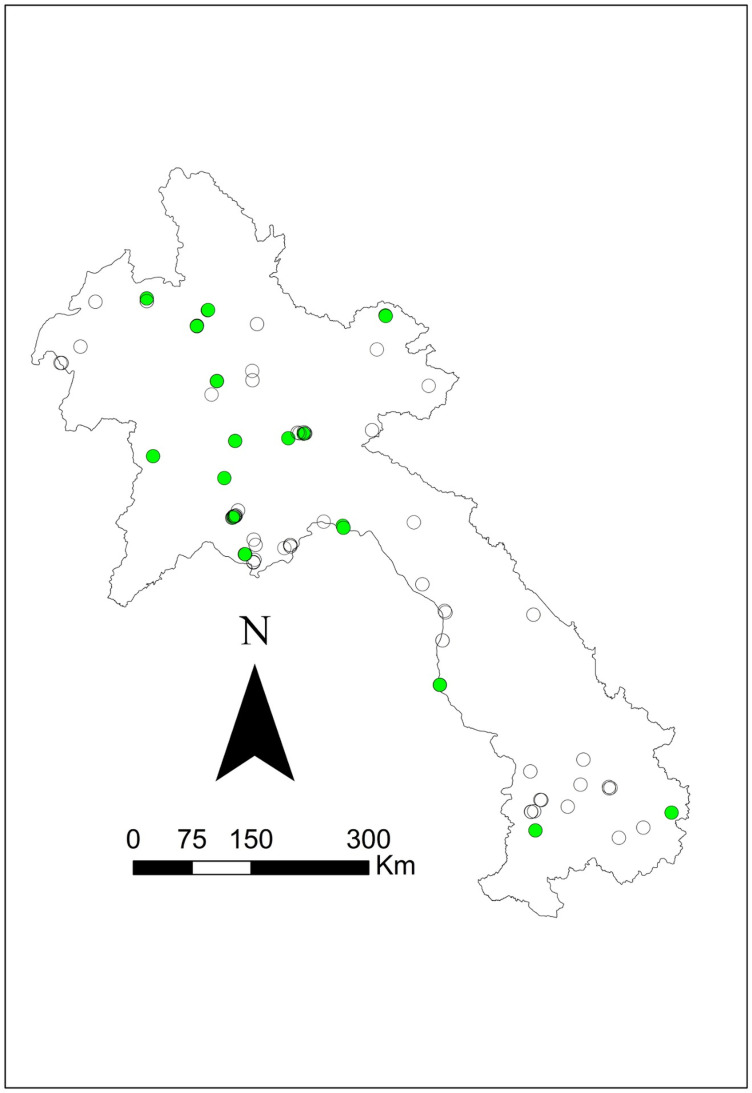
Geographical distribution of *B. pseudomallei* seroprevalence amongst the swine tested using IHA in 2019–2020. Closed green circles represent seropositive, and empty circles represent seronegative. The scale bar and north arrow are included. Map shapefiles were sourced from https://diva-gis.org/data.html.

**Fig 3:**
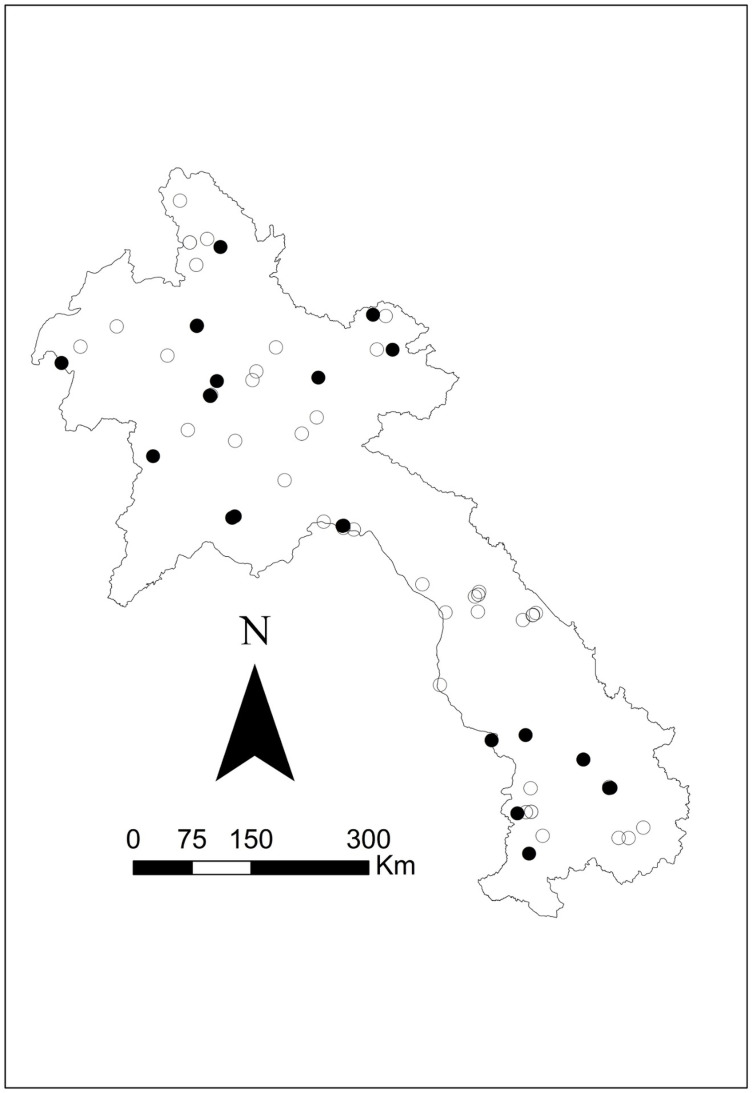
Geographical distribution of *B. pseudomallei* seroprevalence amongst the buffalo tested using IHA in 2019–2020. Closed black circles represent seropositive, and open circles represent seronegative. The scale bar and north arrow are included. Map shapefiles were sourced from https://diva-gis.org/data.html.

**Fig 4:**
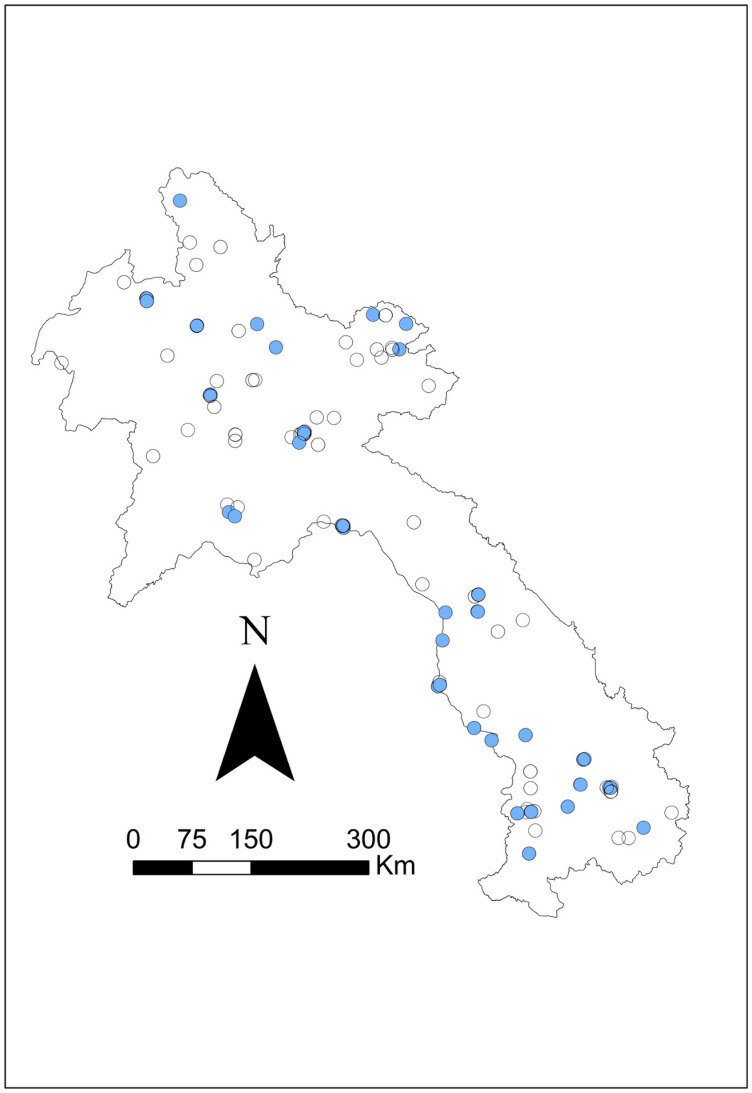
Geographical distribution of *B. pseudomallei* seroprevalence amongst the cattle tested using IHA in 2019–2020. Closed blue circles represent seropositive, and open circles represent seronegative. The scale bar and north arrow are included. Map shapefiles were sourced from https://diva-gis.org/data.html.

**Fig 5:**
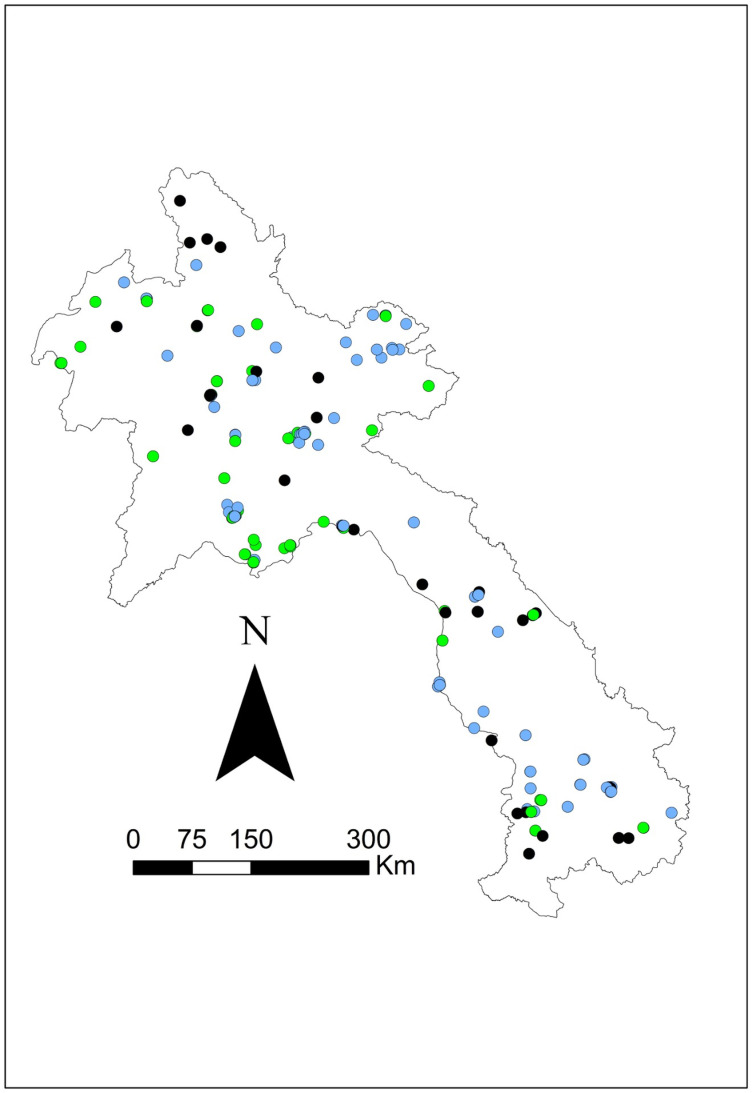
Geographical distribution of all *B. pseudomallei* seropositive amongst the livestock species (swine, buffalo, and cattle) tested using IHA in 2019–2020. Green circles represent swine, black circles represent buffalo, and blue circles represent cattle. The scale bar and north arrow are included. Map shapefiles were sourced from https://diva-gis.org/data.html.

Seroprevalence was mapped using the corresponding latitude and longitude via ArcGIS v10.5 mapping software (ESRI, Redlands, CA) to visually present the data. Symbol maps were created by importing coordinates and seroprevalence estimates and displaying them on a Laos national shapefile. Four additional maps were created: one species-specific map showing *B. pseudomallei* seropositive results for all three species (swine, buffalo, and cattle) and three separate maps displaying melioidosis seropositive results for each species (see Figs 1–5).

### 2.7 Data handling, statistical and spatial analysis

Microsoft Excel 2023 (Microsoft Excel for Microsoft 365 MSO Version 2406) was used for data analysis. The number and percentage of positive and negative *B. pseudomallei* cases for each province and species were determined using the filter, sum and countif functions. Confidence intervals were estimated assuming a binomial distribution. The data were also scanned for spatial clusters of seropositivity using the scan statistic, SaTScan v9.6 [18]. A Bernoulli (case-control) model was used in which seropositive results were cases and seronegative results were controls. A circular spatial scanning window of up to 50% of the study area was applied. The statistical significance of clusters identified was determined through Monte Carlo hypothesis testing by performing 999 Monte Carlo replications and comparing the rank of the maximum likelihood of the field data set with the maximum likelihoods from the random data sets [18]. Clusters were interpreted based on the observed versus expected number of seropositive samples, and cluster locations and size were mapped.

## 3. Results

### 3.1 Number of samples per species analysed

The total number of each species sampled is shown (Table 1). From the 5,190 stored sera available, 917 were selected using simple systematic sampling. This included 499 swine, 281 cattle and 137 buffalo from 16 out of 17 provinces in Lao PDR (Attapeu, Bokeo, Borikhamxay, Champasack, Huaphanh, Khammuane, Luangnamtha, Oudomxay, Phongsaly, Saravane, Savannakhet, Vientiane, Xayaboury, Xaysomboon, Sekong and Xiengkhuang). The sample size allowed an expected overall prevalence of 10% to be estimated with a precision of 2% [19].

**Table 1:** Summary of *B. pseudomallei* seroprevalence for each species, including the number of examined sera, number of *B. pseudomallei* seropositive and % seroprevalence.

Species	Examined sera (n)	*B. pseudomallei* seropositive (n)	*B. pseudomallei* seropositive (%)
Swine	499	20	4.0
Buffalo	137	27	19.7
Cattle	281	64	22.8
Total	917	111	12.1

### 3.2 *B. pseudomallei* seroprevalence

The 917 sera were tested for antibodies against *B. pseudomallei* using the Indirect Haemagglutination Assay in 2019–2020. The IHA *B. pseudomallei* seroprevalence was 4.0% (20/499; 95% C.I. 2.5–6.1%), 19.7% (27/137; 95% C.I. 13.4–27.4%) and 22.8% (64/281; 95% C.I. 18.0–28.1%)) for swine, buffalo and cattle, respectively (Table 1). Of all examined sera, 12.1% (111/917; 95% C.I. 10.1–14.4%) were seropositive. Furthermore, there was a mean of 12.4% *B. pseudomallei* seroprevalence amongst the 16 provinces where samples were collected.

**Fig 6:**
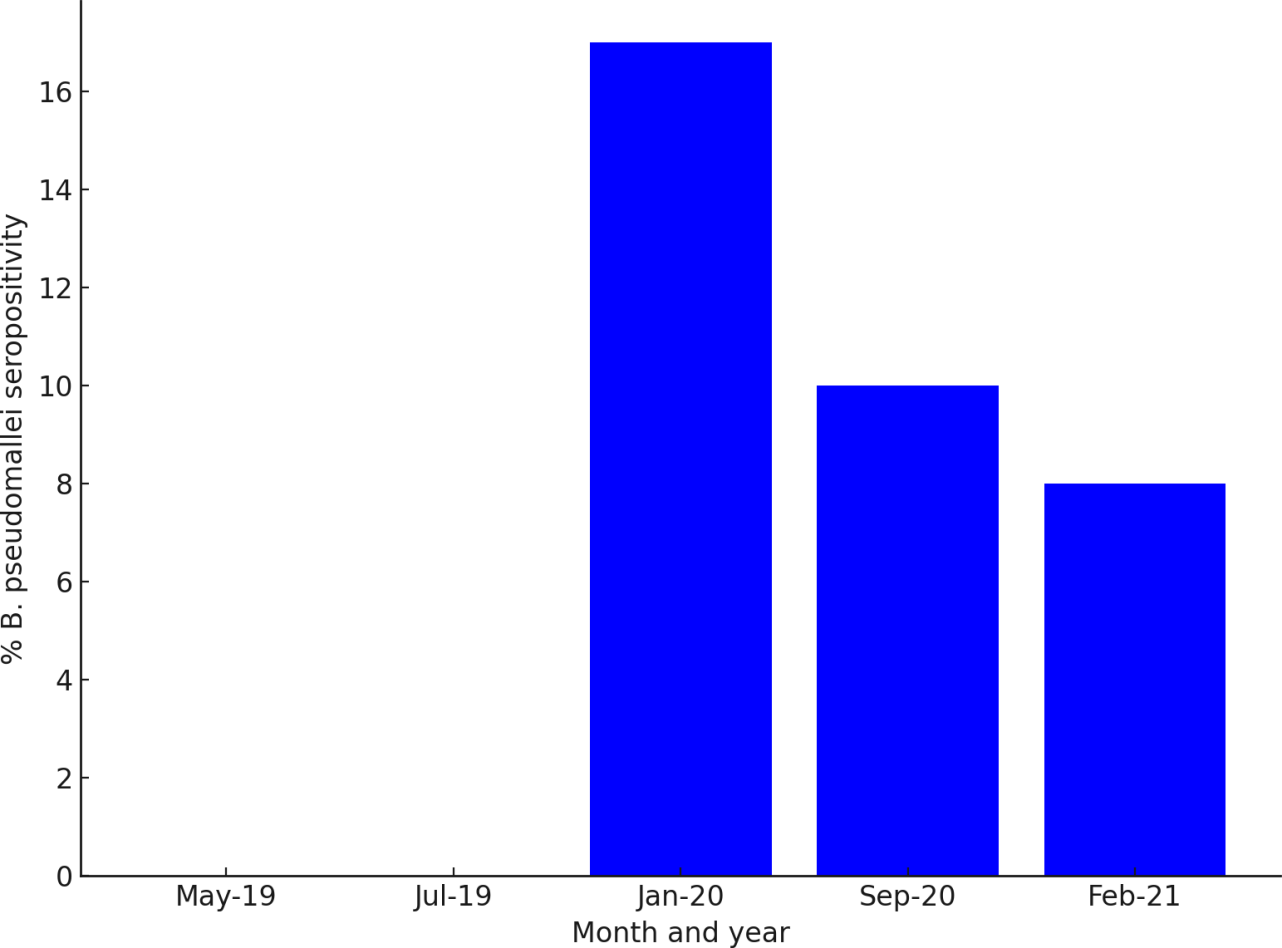
Histogram demonstrating the *B. pseudomallei* % seroprevalence in each serum collection month (May 2019, July 2019, January 2020, September 2020, and February 2021).

Out of the 617 samples collected in May, July and September during the wet season in Laos, 76 samples were *B. pseudomallei* seropositive (12.3%) (Table 2). The remaining 300 samples, collected outside of the wet season, had a seroprevalence of 11.7% (Table 2). January 2020 had the highest percentage of *B. pseudomallei* seropositivity at 17.0% (Table 2 and Fig 6).

**Table 2:** Summary of *B. pseudomallei* seroprevalence for each serum collection month, including the total number of serum samples collected, the number of seropositive and seronegative using IHA, and the calculated percentage of *B. pseudomallei* seropositive.

Collection month	Total (n)	Seropositive (n)	Seroprevalence (%)
May 2019	28	0	0.0
July 2019	24	0	0.0
Jan 2020	118	20	17.0
Sep 2020	229	22	9.6
Feb 2021	182	15	8.2

### 3.3 *B. pseudomallei* geographical distribution

Out of the 16 provinces from which samples were collected, Saravane had the highest seroprevalence (29.4%; 10/34), followed by Sekong (22.7%; 5/22) and Huaphanh (21.6%; 8/37) (Table 3). Fig 1 shows that livestock were exposed to *B. pseudomallei* nationwide. More livestock were exposed in the northern central and southwest regions and occasionally in the northeast region. There was less exposure in the central and the far north regions. More swine were exposed to *B. pseudomallei* in the northern central areas (Fig 2). Livestock were exposed to *B. pseudomallei* across the northern region, and a concentrated buffalo positivity was noted in the southwest region (Fig 3). Similarly, many cattle were exposed within the northern region, with a focused area in the southwest (Fig 4). One significant (p = 0.041) cluster of seropositivity was identified in southwest Laos, adjacent to the Thai border (16.572049°N, 104.768658°E; Kaisone district, Savannakhet province). Four positive samples (one per village location) were detected at this location, with 0.52 expected (observed/expected 7.64). No clusters were detected in the species-specific data.

**Table 3:** The number of *B. pseudomallei* seropositive and seronegative and the *B. pseudomallei* % prevalence for each of the 16 provinces in Laos where samples were collected. Note: There were no samples from Luang Prabang Province.

Province		sero Positive (n)	Total (n)	Seroprevalence (%)
Attapeu	Swine	1	12	8.3
	Buffalo	0	3	0.0
	Cattle	1	5	20.0
	**Total**	**2**	**20**	**10.0**
Bokeo	Swine	0	28	0.0
	Buffalo	1	4	25.0
	Cattle	0	1	0.0
	**Total**	**1**	**33**	**3.0**
Borikhamxay	Swine	2	30	6.7
	Buffalo	2	11	18.2
	Cattle	4	20	20.0
	**Total**	**8**	**61**	**13.1**
Champasack	Swine	1	108	0.9
	Buffalo	4	22	18.2
	Cattle	12	34	35.3
	**Total**	**17**	**164**	**10.4**
Huaphanh	Swine	2	11	18.2
	Buffalo	3	7	42.9
	Cattle	3	19	15.8
	**Total**	**8**	**37**	**21.6**
Khammuane	Swine	0	22	0.0
	Buffalo	0	12	0.0
	Cattle	5	18	27.8
	**Total**	**5**	**52**	**9.6**
Luangnamtha	Swine	1	25	4.0
	Buffalo	0	2	0.0
	Cattle	4	13	30.8
	**Total**	**5**	**40**	**12.5**
Oudomxay	Swine	3	16	18.8
	Buffalo	1	16	6.3
	Cattle	2	17	11.8
	**Total**	**6**	**49**	**12.2**
Phongsaly	Swine	/	/	/
	Buffalo	2	16	12.5
	Cattle	0	11	0.0
	**Total**	**2**	**27**	**7.4**
Saravane	Swine	/	/	/
	Buffalo	4	11	36.4
	Cattle	6	23	26.1
	**Total**	**10**	**34**	**29.4**
Savannakhet	Swine	2	47	4.3
	Buffalo	2	5	40.0
	Cattle	16	52	30.8
	**Total**	**20**	**104**	**19.2**
Vientiane	Swine	4	73	5.5
	Buffalo	2	4	50.0
	Cattle	2	5	40.0
	**Total**	**8**	**82**	**9.8**
Xayaboury	Swine	1	13	7.7
	Buffalo	1	1	100.0
	Cattle	0	4	0.0
	**Total**	**2**	**18**	**11.1**
Xaysomboon	Swine	/	/	/
	Buffalo	0	1	0.0
	Cattle	/	/	/
	**Total**	**0**	**1**	**0.0**
Sekong	Swine	0	7	0.0
	Buffalo	2	5	40.0
	Cattle	3	10	30.0
	**Total**	**5**	**22**	**22.7**
Xiengkhuang	Swine	2	33	6.1
	Buffalo	0	2	0.0
	Cattle	2	24	8.3
	**Total**	**4**	**59**	**6.8**

## 4. Discussion

The major findings of this study are a) animals from the 16 provinces of Laos had been exposed to *B. pseudomallei* with a significant cluster (p = 0.041) in the south-western border adjoining north-eastern Thailand, in the province of Savannakhet, b) cattle had the highest *B. pseudomallei* seroprevalence (22.8%), and c) sera collected in January 2020 had the highest *B. pseudomallei* seroprevalence (17%). Furthermore, no *B. pseudomallei* seroprevalence was recorded in Xaysomboon province, and the sera collected in May and July 2019 were all *B. pseudomallei* seronegative. No seasonal association was found in the seroprevalence data.

Due to the saprophytic nature of *B. pseudomallei* and its persistence and adaptability to soil of 4−42°C, surface water with pH 5−8 and water content of 10−15% [8,20,21], this bacterium thrives in stagnant water in ponds and rice paddies and oil palm tree plantation soil [8] in hot and humid regions, as found in parts of Laos. The highest *B. pseudomallei* seroprevalence in this study was noted in Savannakhet, Saravane and Champasack provinces. This is the first report of *B. pseudomallei* seroprevalence in animals in Champasack. Both Savannakhet and Saravane have been reported as provinces with high *B. pseudomallei* incidence in humans [13]. It has also been demonstrated that Saravane has areas of high *B. pseudomallei* contamination, which is −the highest bacterial soil density ever reported [22]. All three provinces are located in southern Laos, where *B. pseudomallei* is commonly found in the soil and surface waters [22]. During the rainy season, soil erosion containing *B. pseudomallei* drains into the Mekong River along the southwestern border of Laos [23]. The southwestern border also adjoins northeastern Thailand, where the bacterium has been found in 50% of soil samples [22] and has the highest estimated *B. pseudomallei* seroprevalence [1,3,7].

In comparison to southern Laos, *B. pseudomallei* seroprevalence was lower in northern Laos. In a study investigating river water samples, Zimmermann et al. [24] also found an absence of *B. pseudomallei* in the Northern Highlands. While the geographical variation could not be definitively explained, this might be due to differences in environmental factors such as climate, soil types and land use [24]. A correlation has been reported between local rainfall and clusters of *B. pseudomallei* cases, in which 50−75% are present during the rainy season [8,10]. Although our results are not consistent with those findings, it could be possible that 2019–2020 had less extreme weather events and a prolonged dry season; flooding and typhoons can reactivate the latent population of *B. pseudomallei* [25], and UV exposure and dryness can limit its distribution [22]. Data from confirmed cases of melioidosis in humans show a similar geographical distribution, with a predominance in the south of Laos [13]. This suggests that surveillance of melioidosis in livestock might act as a sentinel for human disease risk.

Among this study’s three livestock species, cattle and buffalo had the highest seroprevalence (~20%), and swine had the lowest *B. pseudomallei* seroprevalence (4.0%). Similar findings were reported in previous studies – 22% seroprevalence in dairy cattle in the northeastern region of Thailand [5] and 6.1% seropositive swine in three provinces of Vietnam [26]. Limmathurotsakul et al. [3] estimated an incidence rate of 0.02% and 0.01% for swine and cattle, respectively. However, their analysis only included animals with *B. pseudomallei* as the cause of death that were both IHA and culture-positive [3]. Our findings support other studies in the region suggesting that cattle and buffalo are more commonly exposed to *B. pseudomallei* than swine.

One of the major limitations of this study is that information on whether the collected sera were from animals sent for slaughter for sale or salvage value due to potential *B. pseudomallei* infections was unavailable. Additionally, it has been reported that the major signs of *B. pseudomallei* infection in swine are subclinical [2], and adult pigs are often presented with chronic infections [26]. Therefore, it is likely that the swine seroprevalence estimates we have reported here are underestimated. Geographically, Champasack only had one *B. pseudomallei* seropositive swine sera out of the 108 sera collected from that province. In contrast, Champasack had high numbers of *B. pseudomallei* seropositive for both buffalo (4/27) and cattle (12/64) (Table 3). Regardless, the difference between cattle/buffalo and swine seroprevalence could be due to the different production systems: grazing animals have greater exposure to environmental *B. pseudomallei* compared to animals in pens (Norris et al., 2020) [26], hence the higher *B. pseudomallei* seroprevalence we estimated for the grazing animal species in this study – buffalo (19.7%) and cattle (22.8%). Grazing animals encounter greater exposure to environmental *B. pseudomallei* than animals in enclosed housings, however the difference in their susceptibility to *B. pseudomallei* infection could not be analysed in this study.

Other limitations of this study include the difficulty of analysing data at the village level and the resource-limited setting, which could have affected the accuracy of the collected data. The sampling frame used in the study was from an abattoir serum surveillance project, which could be biased towards the types of livestock selected for slaughter. Furthermore, the total number of samples tested reflected the availability of test kits. However, efforts were made to include randomisation in the selection of samples for testing. Data on animals that have died of melioidosis or other unknown causes could be obtained from veterinary research centres and the Ministry of Agriculture and Forestry of Laos in the future for further geographic visualisation and analysis, although Rattanavong et al. [22] reported that there is only one veterinary laboratory with routine and accessible diagnostic service for *B. pseudomallei* in the capital city Vientiane. Another limitation is the presence of IHA false positive test results, as healthy animals can have high bacterial titres in melioidosis-endemic areas [4].

Furthermore, exposure to other closely related *Burkholderia spp.* may lead to antibodies against *B. pseudomallei* [27]. In future studies, it is recommended that culture of blood or pus samples for *B. pseudomallei* should be performed in conjunction with IHA [3] since IHA alone is insufficient for defining exposure [14]. It has been reported that IgM ELISA has higher sensitivity and specificity, 88% and 92.2%, respectively [28]. An even higher specificity and sensitivity (95.4% and 100%, respectively) can be achieved when IHA is combined with IgM ELISA [28]; however, the cost is higher [29]. One challenge is that most tests targeting *B. pseudomallei* were developed for diagnosis rather than exposure, and there is no gold standard diagnostic test for exposure to *B. pseudomallei*.

Our study revealed the location of a significant *B. pseudomallei* cluster in Laos, together with regional variations, which will be helpful for future prevention and monitoring of this disease in livestock and potentially in humans. The concept of using livestock surveillance for supporting risk management for human infections could also be further explored in a One Health context. Examining both human and livestock surveillance temporal and spatial patterns may support better preventive management for public health. Future studies should focus on the different susceptibilities to melioidosis amongst different livestock species and the potential risk factors, specifically regarding livestock farming activities, livestock density, land-use patterns, and environmental risk factors.

## References

[1] Birnie E, Biemond JJ, Wiersinga WJ. Drivers of melioidosis endemicity: epidemiological transition, zoonosis, and climate change. Curr Opin Infect Dis. 2022;35(3):196–204. doi: 10.1097/QCO.0000000000000827 35665713 PMC10128909

[2] Kwanhian W, Jiranantasak T, Kessler AT, Tolchinsky BE, Parker S, Songsri J, et al. Investigation of melioidosis outbreak in pig farms in southern Thailand. Vet Sci. 2020;7(1):9. doi: 10.3390/vetsci7010009 31947512 PMC7157537

[3] Limmathurotsakul D, Thammasart S, Warrasuth N, Thapanagulsak P, Jatapai A, Pengreungrojanachai V, et al. Melioidosis in animals, Thailand, 2006–2010. Emerg Infect Dis. 2012;18(2):325–7. doi: 10.3201/eid1802.111347 22304782 PMC3310465

[4] Srikawkheaw N, Lawhavinit O. Detection of antibodies against melioidosis from animal sera in Thailand by indirect haemagglutination test. Agric Nat Resour. 2007;41(5):81–5.

[5] Srikitjakarn L, Sirimalaisuwan A, Khattiya R, Krueasukhon K, Mekaprateep M. Seroprevalence of melioidosis in dairy cattle in Chiang Mai Province, northern Thailand. Southeast Asian J Trop Med Public Health. 2002;33(4):739–41. 12757220

[6] Perumal Samy R, Stiles BG, Sethi G, Lim LHK. Melioidosis: clinical impact and public health threat in the tropics. PLoS Negl Trop Dis. 2017;11(5):e0004738. doi: 10.1371/journal.pntd.0004738 28493905 PMC5426594

[7] Cheng AC, Currie BJ. Melioidosis: epidemiology, pathophysiology, and management. Clin Microbiol Rev. 2005;18(2):383–416. doi: 10.1128/CMR.18.2.383-416.2005 15831829 PMC1082802

[8] Buisson Y, Rattanavong S, Keoluangkhot V, Vongphayloth K, Manivanh L, Phetsouvanh R, et al. Melioidosis in Laos. In: Morand S, Dujardin JP, Lefait-Robin R, Apiwathnasorn C, editors. Socio-ecological dimensions of infectious diseases in Southeast Asia. Singapore: Springer; 2015. p. 89–104.

[9] Dance DA. Melioidosis as an emerging global problem. Acta Trop. 2000;74(2–3):115–9. doi: 10.1016/s0001-706x(99)00059-5 10674638

[10] Kaestli M, Mayo M, Harrington G, Ward L, Watt F, Hill JV, et al. Landscape changes influence the occurrence of the melioidosis bacterium *Burkholderia pseudomallei* in soil in northern Australia. PLoS Negl Trop Dis. 2009;3(1):e364. doi: 10.1371/journal.pntd.0000364 19156200 PMC2617783

[11] Millan JM, Mayo M, Gal D, Janmaat A, Currie BJ. Clinical variation in melioidosis in pigs with clonal infection following possible environmental contamination from bore water. Vet J. 2007;174(1):200–2. doi: 10.1016/j.tvjl.2006.05.006 16807011

[12] Choy JL, Mayo M, Janmaat A, Currie BJ. Animal melioidosis in Australia. Acta Trop. 2000;74(2–3):153–8. doi: 10.1016/s0001-706x(99)00065-0 10674644

[13] Dance DAB, Luangraj M, Rattanavong S, Sithivong N, Vongnalaysane O, Vongsouvath M, et al. Melioidosis in the Lao People’s Democratic Republic. Trop Med Infect Dis. 2018;3(1):21. doi: 10.3390/tropicalmed3010021 30274419 PMC6136615

[14] Chaichana P, Jenjaroen K, Amornchai P, Chumseng S, Langla S, Rongkard P, et al. Antibodies in melioidosis: the role of the indirect hemagglutination assay in evaluating patients and exposed populations. Am J Trop Med Hyg. 2018;99(6):1378–85. doi: 10.4269/ajtmh.17-0998 30298810 PMC6283516

[15] Harris PNA, Ketheesan N, Owens L, Norton RE. Clinical features that affect indirect-hemagglutination-assay responses to *Burkholderia pseudomallei*. Clin Vaccine Immunol. 2009;16(6):924–30. doi: 10.1128/CVI.00026-09 19403784 PMC2691047

[16] Wongratanacheewi S, Sermswan RW, Anuntagool N, Sirisinha S. Retrospective study on the diagnostic value of IgG ELISA, dot immunoassay and indirect hemagglutination in septicemic melioidosis. Asian Pac J Allergy Immunol. 2001;19(2):129–33. 11699719

[17] Wiersinga WJ, Birnie E, Weehuizen TA, Alabi AS, Huson MA, Huis RA, et al. Clinical, environmental, and serologic surveillance studies of melioidosis in Gabon, 2012–2013. Emerg Infect Dis. 2015;21(1):40–7. 25530077 10.3201/eid2101.140762 PMC4285261

[18] Kulldorff M. A spatial scan statistic. Commun Stat Theor Methods. 1997;26(6):1481–96. doi: 10.1080/03610929708831995

[19] Ausvet. Epitools - epidemiological calculators 2025 [cited 2025 Jan 29]. Available from: https://epitools.ausvet.com.au

[20] Chen YS, Chen SC, Kao CM, Chen YL. Effects of soil pH, temperature and water content on the growth of *Burkholderia pseudomallei*. Folia Microbiol (Praha). 2003;48(2):253–6. doi: 10.1007/BF02930965 12800512

[21] Palasatien S, Lertsirivorakul R, Royros P, Wongratanacheewin S, Sermswan RW. Soil physicochemical properties related to the presence of *Burkholderia pseudomallei*. Trans R Soc Trop Med Hyg. 2008;102(Suppl 1):S5–9. doi: 10.1016/s0035-9203(08)70003-8 19121688

[22] Rattanavong S, Wuthiekanun V, Langla S, Amornchai P, Sirisouk J, Phetsouvanh R, et al. Randomized soil survey of the distribution of *Burkholderia pseudomallei* in rice fields in Laos. Appl Environ Microbiol. 2011;77(2):532–6. doi: 10.1128/AEM.01822-10 21075883 PMC3020526

[23] Hassan AKR, Inglis TJJ, O’Rilley L, Ooi CH, Bohari H. Environmental surveillance for potential human exposure to *Burkholderia pseudomallei* causing melioidosis in changing land use in East Malaysia. Int J Sci Basic Appl Res. 2015;22:263–73.

[24] Zimmermann RE, Ribolzi O, Pierret A, Rattanavong S, Robinson MT, Newton PN, et al. Rivers as carriers and potential sentinels for *Burkholderia pseudomallei* in Laos. Sci Rep. 2018;8(1):8674. doi: 10.1038/s41598-018-26684-y 29875361 PMC5989208

[25] Merritt AJ, Inglis TJJ. The role of climate in the epidemiology of melioidosis. Curr Trop Med Rep. 2017;4(4):185–91. doi: 10.1007/s40475-017-0124-4 29188170 PMC5684260

[26] Norris MH, Tran HTT, Walker MA, Bluhm AP, Zincke D, Trung TT, et al. Distribution of serological response to *Burkholderia pseudomallei* in swine from three Provinces of Vietnam. Int J Environ Res Public Health. 2020;17(14):5203. doi: 10.3390/ijerph17145203 32708490 PMC7399857

[27] Rongkard P, Kronsteiner B, Hantrakun V, Jenjaroen K, Sumonwiriya M, Chaichana P, et al. Human immune responses to melioidosis and cross-reactivity to low-virulence Burkholderia species, Thailand. Emerg Infect Dis. 2020;26(3):463–71. 32091359 10.3201/eid2603.190206 PMC7045851

[28] Kunakorn M, Boonma P, Khupulsup K, Petchclai B. Enzyme-linked immunosorbent assay for immunoglobulin M specific antibody for the diagnosis of melioidosis. J Clin Microbiol. 1990;28(6):1249–53. doi: 10.1128/jcm.28.6.1249-1253.1990 2199494 PMC267913

[29] Dhanalakshmi S, Meenachi C, Parija SC. Indirect haemagglutination test in comparison with ELISA for detection of antibodies against invasive amoebiasis. J Clin Diagn Res. 2016;10(8):DC05–8. doi: 10.7860/JCDR/2016/21566.8326 27656436 PMC5028449

